# MDF-DTA: A Multi-Dimensional Fusion Approach for Drug-Target Binding Affinity Prediction

**DOI:** 10.1021/acs.jcim.4c00310

**Published:** 2024-06-18

**Authors:** Amit Ranjan, Adam Bess, Chris Alvin, Supratik Mukhopadhyay

**Affiliations:** † Department of Environmental Sciences, Louisiana State University, Baton Rouge, Louisiana 70803, United States; ‡ Department of Computer Science, Furman University, Greenville, South Carolina 29613, United States

## Abstract

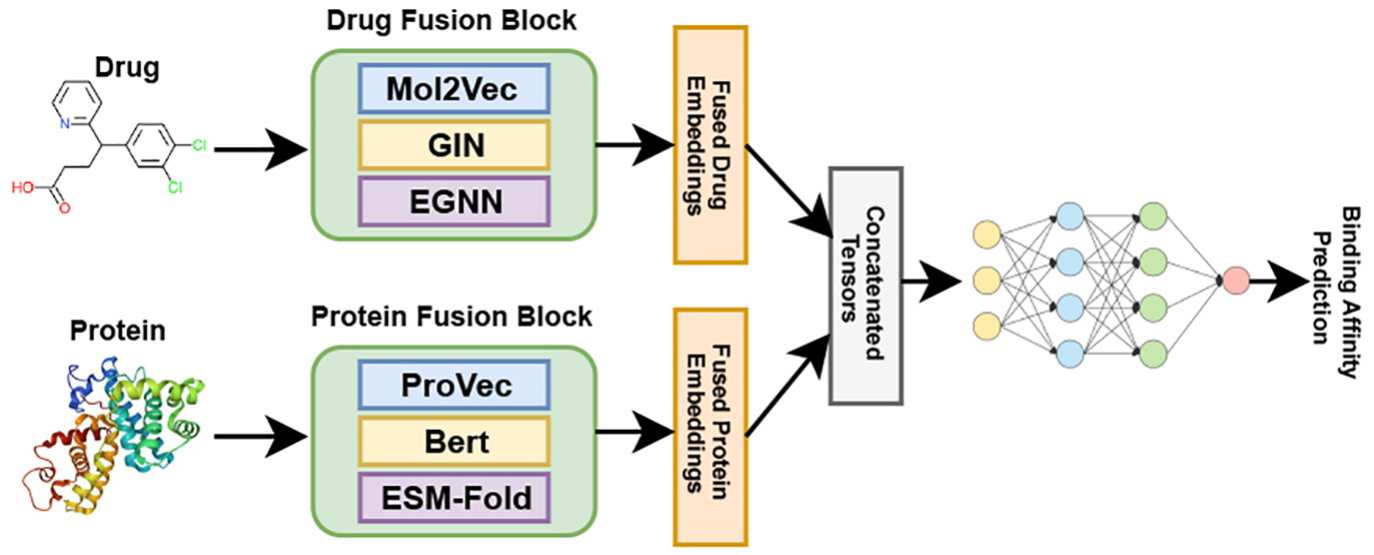

Drug-target affinity (DTA) prediction is an important task in the early stages of drug discovery. Traditional biological approaches are time-consuming, effort-consuming, and resource-consuming due to the large size of genomic and chemical spaces. Computational approaches using machine learning have emerged to narrow down the drug candidate search space. However, most of these prediction models focus on single feature encoding of drugs and targets, ignoring the importance of integrating different dimensions of these features. We propose a deep learning-based approach called Multi-Dimensional Fusion for Drug Target Affinity Prediction (MDF-DTA) incorporating different dimensional features. Our model fuses 1D, 2D, and 3D representations obtained from different pretrained models for both drugs and targets. We evaluated MDF-DTA on two standard benchmark data sets: DAVIS and KIBA. Experimental results show that MDF-DTA outperforms many state-of-the-art techniques in the DTA task across both data sets. Through ablation studies and performance evaluation metrics, we evaluate the importance of individual representations and the impact of each representation on MDF-DTA.

## Introduction

Discovering new drugs requires a significant investment of time and money.^1^ The basis of this complex process is identifying drugs that may interact with particular protein targets. This process is essential for both the discovery of novel drugs as well as the repurposing of currently available drugs and the anticipation of their potential side effects.^1^ However, the enormous number of chemical properties that must be considered while investigating potential drug candidates against a target protein makes the drug discovery process challenging.^2^

In drug discovery, there are typically two primary ways that computational methods help us predict how drugs interact with particular targets: the molecular docking-based approach and the computational approach.^3,4^ To predict drug-target interaction (DTI), the docking-based approach focuses on the 3D structures of proteins. However, this approach has its own limitations. The method is generally slow and computationally expensive.^5^ When dealing with novel proteins with structures that researchers do not know, this approach also runs into problems. Even if we spend an enormous amount of time using similarity models to predict the molecular structure of the protein, we might not have accurate structural data.^4^ Given these limitations, a computational approach can be considered an acceptable alternative for molecular docking. This approach includes different models, which can be classified into two types: statistical machine-learning models and deep-learning models, which predict DTA by investigating the representations of drug molecules and target proteins.^6^

Statistical machine learning-based computational approaches serve as a basis for predicting drug-target interactions.^3^ In this approach, the problem is frequently presented as a dichotomous categorization problem wherein the model decides whether a drug can bind together with a target or not. Recently, there has been a trend toward predicting continuous binding affinity values using regression.^6^ By applying different feature selection and extraction algorithms, these developments improve prediction accuracy. The idea is to feed these extracted features into conventional statistical machine learning models to improve the prediction accuracy of the affinity score between drugs and target proteins. Therefore, the model’s architecture used to train these representations and the formulation of the input features are crucial factors determining how well the prediction model performs. To predict the binding affinities of drug-target pairings, two well-known statistical machine-learning algorithms that achieved remarkable performance are KronRLS^7^ and SimBoost.^8^ Using the Kronecker Regularized Least Squares technique, KronRLS^7^ leverages knowledge of drug chemical structure and similarity score of target sequences. Although it is excellent at predicting continuous affinity values, its linear approach prevents it from accurately capturing complicated, nonlinear connections. SimBoost,^8^ on the other hand, adopts a different strategy by taking into account network-based interaction aspects as well as similarities between drugs and targets. For prediction, it uses a gradient-boosting algorithm. SimBoost relies on evolutionary-based data for proteins and 2D representations for drugs despite its different knowledge sources.

Our MDF-DTA approach attempts to address the aforementioned challenges of computational efficiency, cost-effectiveness, and improved prediction accuracy in DTA prediction. These approaches use protein and drug sequences to extract highly informative features.

One such deep learning-based method, DeepDTA,^9^ uses a Convolutional Neural Network (CNN) based architecture to extract features from protein sequences and SMILES^10^ sequences. To encode proteins and drugs, this method makes use of Smith-Waterman^11^ and CNN-based features. DeepDTA^9^ has been trained on a relatively small collection of labeled sequence data, which can limit its performance despite its strengths in autonomous feature extraction. WideDTA^12^ expanded the search for richer data sources by including additional forms of sequence data such as protein domains, motifs, and ligand maximal common substructures. Although this method improves prediction accuracy, it does so at the cost of the network’s overall complexity because it requires a larger input data configuration and more CNN blocks. Researchers have also developed AttentionDTA^13^ and MATT-DTI,^14^ both of which incorporate additional attention layers in addition to the CNN blocks for more insightful feature extraction. The identified attributes are nonetheless bound by the limited labeled sequence data because these approaches, like their predecessors, rely on sequence-based representations of proteins and SMILES for drugs. Few studies also used variational autoencoders (VAE) to extract molecular sequence features from SMILES and protein sequences, adding interaction paths among sequence pairs and establishing correlations between molecular substructures to predict affinity scores.^15^ These techniques have significant potential for improving predictions by capturing complex ways in which drugs and targets interact. Nevertheless, the availability and quality of labeled sequence data, heavily impact the way these models operate.

Transformer-based approaches emerged as an alternative solution to the issue of limited labeled data for constructing distributed representations in DTA prediction, motivated by their effective utilization in natural language processing (NLP) tasks.^16^ Among these approaches, MT-DTI^17^ and FusionDTA^18^ outperforms other models. In MT-DTI,^17^ CNN blocks are combined with a molecular transformer to encode drug and protein sequences, providing useful distributed representation vectors. In a similar manner, FusionDTA^18^ uses the ESM-Fold^19^ transformer to construct distributed representation vectors from protein sequences, producing encouraging results. To effectively encode protein sequences, FusionDTA needs extra pretraining and fine-tuning stages. Despite these developments, transformer-based techniques require more time and space to create distributed representation vectors. Moreover, these techniques frequently emphasize sequence information during feature extraction, thus missing out on valuable structural characteristics of molecular compounds. Additionally, hybrid-based methods were also introduced to capture the integration of the drug structures into sequence-based techniques. Recently, Zhu et al., proposed a transformer-based diffusion technique to predict the binding affinity score through the use of multiscale feature interaction and graph optimization methodology to improve the model’s performance and interpretability.^20^ A similar study was proposed where transformers were used for target featurization and autoencoders for SMILES featurization, employing adaptive attention pooling to enhance the performance of DTI.^21^

To enhance the extraction of features, particularly in terms of topological information, researchers have introduced graph neural network (GNN) based methods.^22−24^ These methods take advantage of the spatial, sequential, and structural properties of both drugs and proteins. Researchers have proposed employing molecular graph representations of drugs as an alternate strategy to create rich feature representations. For example, GraphDTA^23^ analyzes drugs using atomic features: four GNN layers capture complex graph representations, taking it a step further than previous research in terms of topological information. However, to capture complex local and global features of molecules, GNN-based methods frequently use external cheminformatics libraries and deep layers to extract multihop neighbor encoding. While these factors enhance feature extraction, they also add complexity because of the depth of the models. The motivation behind our study lies within the fact that despite encouraging progress, most of the previous studies rely on only one or two featurizations that might achieve suboptimal performance but overlook the impact of other dimensional features to predict binding affinity. For instance, the 2D structure plays a crucial role in various drug-related aspects like toxicity, whereas the 3D arrangement likely influences properties associated with quantum mechanics, such as single-point energy, atomic forces, or dipole moments. Hence, it is logical to integrate advantages from various representations and weigh the relative merits of each in forecasting binding affinity.

Our MDF-DTA approach attempts to address the aforementioned challenges, including computational efficiency, cost-effectiveness, and prediction accuracy in DTA prediction. MDF-DTA incorporates multidimensional embeddings (i.e., 1D, 2D, and 3D) of drugs and proteins, including sequence, graph, and structure features. This approach builds on the fact that merging information from multiple dimensions enhances the overall predictive power compared to relying on a single dimension.^25^ MDF-DTA holds the promise of significantly improving the efficiency, accuracy, and cost-effectiveness of drug discovery efforts.

**Figure 1:**
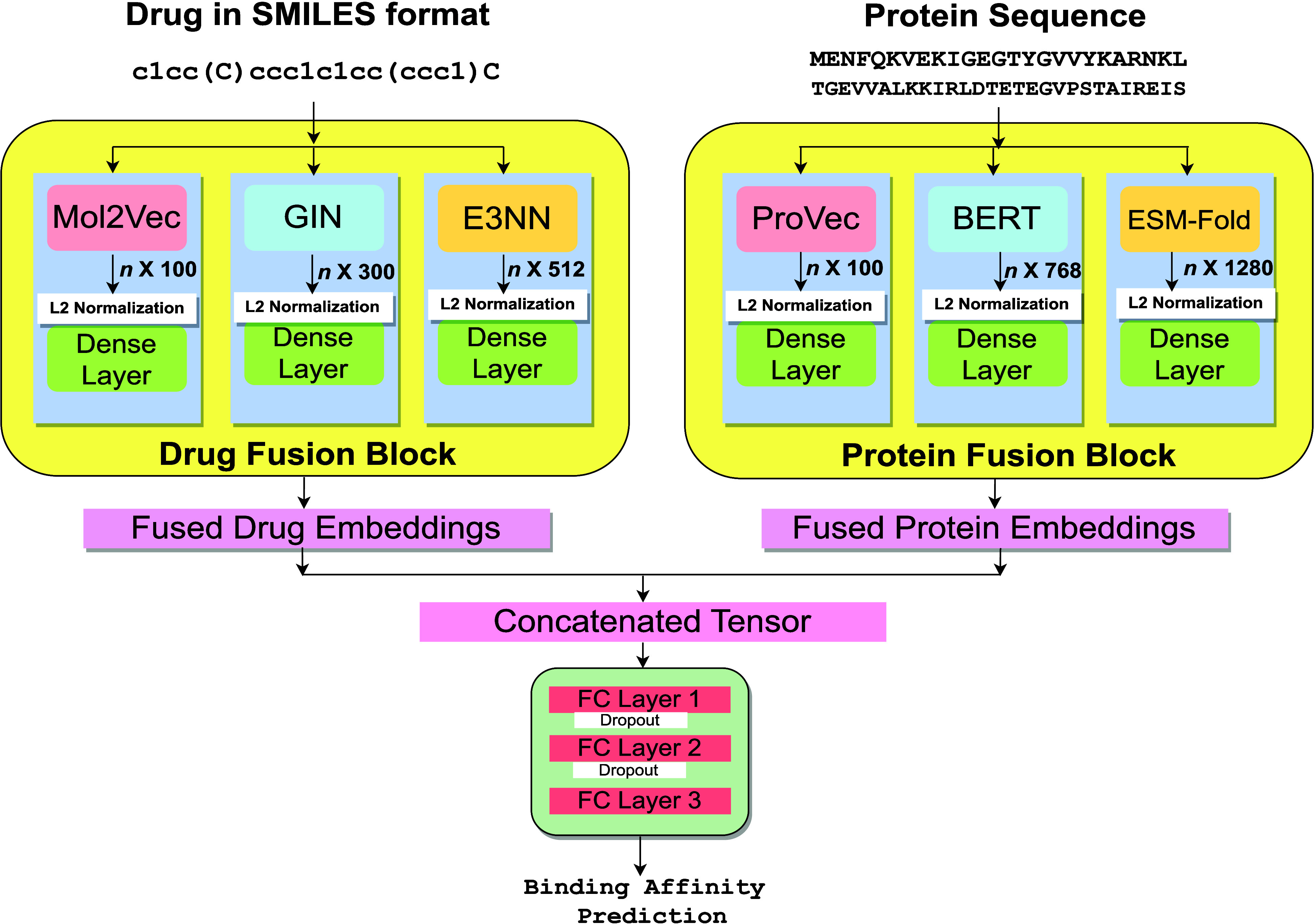
Overview of the MDF-DTA architecture. On the left, drug fusion block performs 1D drug encoding using a Mol2Vec model, 2D graph embeddings using a GIN, and 3D embeddings using an E3NN. On the right, protein fusion block performs 1D protein encoding from ProtVec, 2D embeddings from ProtBERT model, and 3D embeddings using ESM-Fold. After obtaining embeddings in both blocks, L2 normalization is used to ensure consistent scales. Then the normalized embeddings are processed through dense layers, resulting in three different outputs in each block. The outputs of dense layers in drug fusion block are concatenated to obtain fused drug embeddings. Similarly, the outputs of dense layers in protein fusion block are concatenated to obtain the fused protein embeddings. Then both fused embeddings are further concatenated, forming a tensor. The tensor is passed through three fully connected layers with a dropout layer to predict final affinity score.

As shown in Figure 1, our MDF-DTA architecture begins by generating features for drugs and proteins through transfer learning.^26^ The use of transfer learning leverages pretrained models for feature generation for drugs and proteins. The process that we refer to as “fusion” is implemented using two blocks to combine embeddings generated from pretrained models, followed by L2 normalization^27^ and then passing them through a dense layer. As described in greater detail in Section 2, this process is performed for both drug and protein sequences, and thus, we have a drug fusion block and a protein fusion block, each of which combines dimensional embeddings. The outputs of these two blocks are merged to get a concatenated tensor, which is passed through a three-layered fully connected network to predict binding affinity scores.

For drugs, we used a *drug fusion block* to obtain fused drug embeddings. Initially, we generated 1D embeddings from input SMILES using pretrained Mol2Vec model.^28^ Subsequently, we employed a pretrained Graph Isomorphic Network (GIN)^24^ model to acquire 2D embeddings. GIN, a type of Graph Neural Network (GNN), excels at learning 2D features from graphical structures. Additionally, we utilized a pretrained Equivariant Graph Neural Network (EGNN)^29^ model to derive 3D embeddings, capturing features from the spatial arrangement of molecular atoms and residues within SMILES sequences. The three different types of embeddings obtained are then normalized using L2 normalization. The normalized embeddings are further passed through three dense layers, each dedicated to one type of embedding. The outputs of these dense layers are merged to obtain the final fused drug embeddings.

For proteins, we construct a *protein fusion block* similar to how we constructed our drug fusion block. We derive 1D embeddings using the pretrained ProtVec^30^ model, which leverages a CNN to represent the entire protein as a vector. Additionally, we employ a pretrained ProtBERT^31^ model to capture 2D protein embeddings. ProtBERT, based on transformer-based networks, offers richer insights compared to ProtVec. Finally, we incorporate 3D embeddings from the pretrained ESM-Fold^19^ model, which provides even deeper insights into protein 3D structures. These dimensional embeddings are then normalized using L2 normalization and passed through a dense layer. The outputs of dense layers are further combined to obtain the final comprehensive fused protein embeddings.

This paper makes the following contributions:

1. A fusion-based approach to enhance drug-target binding affinity score predictions by aggregating dimensional information from drugs and proteins, including 1D, 2D, and 3D representations.
2. Evaluating the effectiveness of the MDF-DTA for predicting DTA scores, addressing both classification and regression tasks.
3. Conducting systematic analyses through ablation studies to assess the robustness of the proposed framework and identify key contributing factors.
4. Incorporating embedding techniques, such as ProtVec, ProtBERT, ESM-Fold, Mol2Vec, GIN, and ESMFold, to capture multiple dimensional featurization at different levels of abstraction and demonstrating superior predictive performance of MDF-DTA compared to existing state-of-the-art models.

## Materials and Methodology

### Data Sets

We evaluated the performance of MDF-DTA using two standard benchmark data sets that have been previously used for evaluating the effectiveness of computational models in predicting the binding affinities of drug-target pairs: DAVIS^32^ and KIBA^33^ data sets.

#### DAVIS

:^32^ The DAVIS data set consists of 30,056 interactions involving 442 distinct proteins and 68 unique ligands. This data set provides binding affinity measurements in terms of the dissociation constant (*K*_*d*_) values, which signify the potency of the interaction between a drug and its respective target. In the data set, affinity scores are in *K*_*d*_ values. To enhance the interpretability and manage the numerical diversity of *K*_*d*_ values, the authors in^9^ introduced a logarithmic transformation by introducing a novel measure, denoted as *pK*_*d*_. Mathematically, *pK*_*d*_ is obtained as follows:
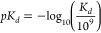
1

**Figure 2:**
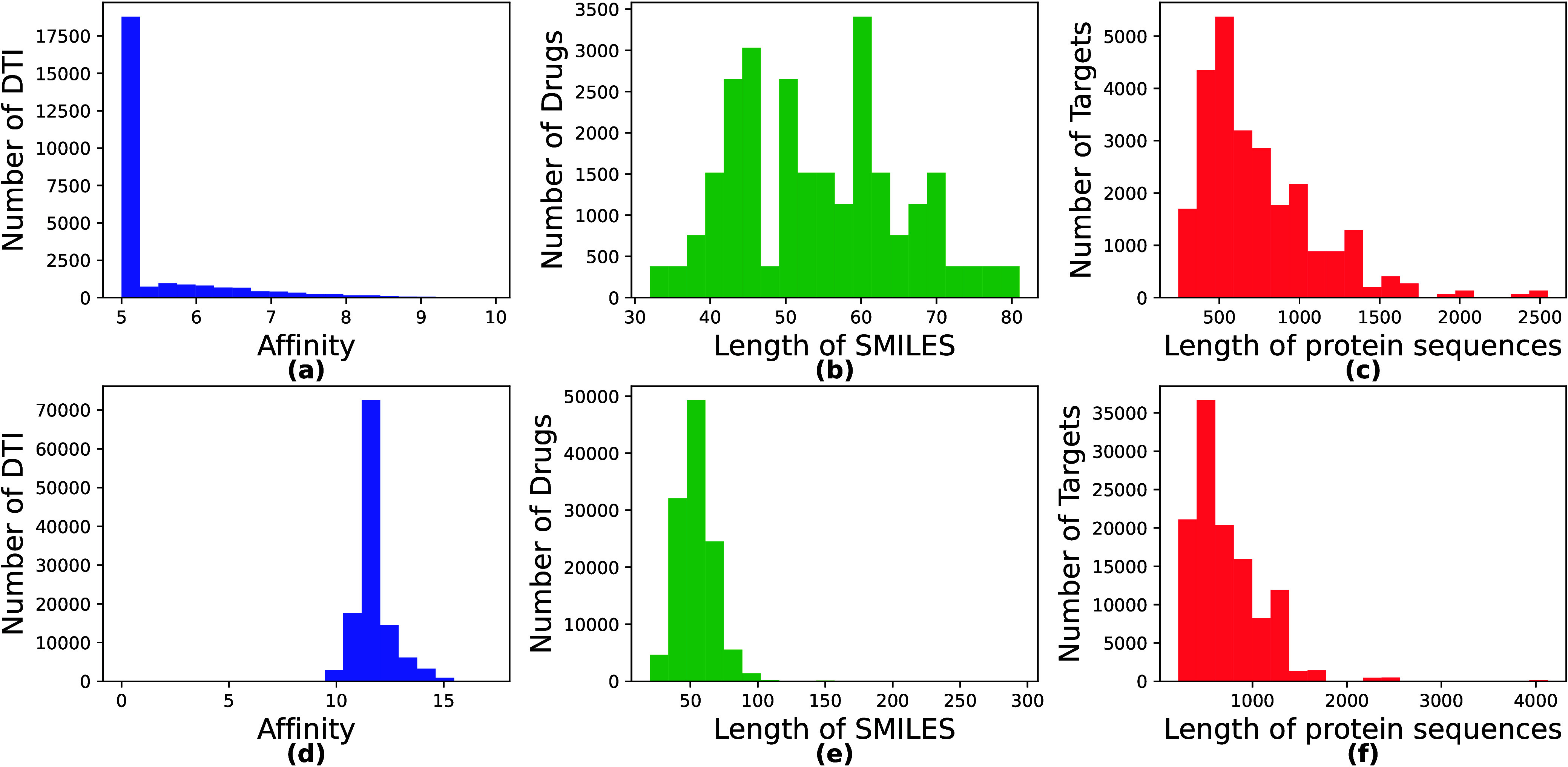
Illustration of DAVIS and KIBA data set data distribution through frequency histograms in which (a) and (d) represent the distributions of binding affinity, (b) and (e) represent the ligand SMILES length, and (c) and (f) represents the protein sequence lengths of DAVIS and KIBA data set, respectively.

A pictorial representation of the histograms of affinity, drug length, and protein length of the DAVIS data set is shown in Figure 2. The range of binding affinity values in the DAVIS data set is between 5 and 10. Also, the protein lengths range from 400 to 1500., with the largest distribution of 500 and a maximum length of 2549. Further analyzing the data set, we found that the distribution of affinity values is concentrated around 5, accounting for more than 50% of the interactions.

#### KIBA:^33^

The KIBA data set contains 118,218 interactions involving 467 proteins and 52,498 ligands, representing a diverse range of drugs and biological targets. The KIBA data set takes an integrated strategy by taking into account several inhibitor efficacy measures, such as *K*_*i*_, *K*_*d*_, and *IC*_50_. In context of KIBA data set, *K*_*i*_ denotes measuring the concentration of kinase inhibitor needed to achieve 50% inhibition of the kinase’s activity, *K*_*d*_, refers to the required concentration of an inhibitor for inhibiting the enzymatic activity, and *IC*_50_ denotes the concentration of the inhibitor at which half of the binding sites on the target kinase are occupied. Histograms of affinity, drug length and protein length of the KIBA data set are shown in Figure 2. We observe that the KIBA data set’s binding affinity values range between 10 and 13, with most interactions centered at an affinity value of 11. The length of target protein sequences is between 200 and 1500 amino acids, with an average length of 700 amino acids.

A significant portion of protein pairs in both the KIBA and DAVIS data sets exhibit low similarity, thus emphasizing the nonredundancy of the data sets. In the DAVIS data set, 92% of protein–protein Smith-Waterman similarities^11^ have no more than 60%, and in the KIBA data set, all but 1% of protein pairs have a similarity of no more than 60%.

### MDF-DTA Architecture

#### Drug Fusion Block

As shown in Figure 1, the left section of MDF-DTA comprises a drug fusion block that encodes drug embeddings through three distinct components, each dedicated to a specific drug embedding. In the first component of drug fusion block, a SMILES sequence representing a drug undergoes processing via a pretrained Mol2Vec model^28^ capturing the sequential features of a molecular compound by generating 1D drug embeddings of length 100. The second component utilizes a GIN network,^24^ trained on a collection of unlabeled drug graphs, to process a drug’s 2D molecular graph, yielding embeddings with an array length of 300, encapsulating graphical features. The third component captures the spatial features of atoms from the SMILES sequence using an EGNN,^29^ resulting in embeddings of length of 512. Following the extraction of all three embeddings types, L2 normalization^27^ is employed to normalize all embeddings in each component, ensuring consistent feature scales. This normalization technique converts the embeddings into unit vectors. Moreover, input layers were established for each component to receive the drug embeddings represented in 1-dimensional arrays. Each input passes through a fully connected layer consisting of 1024 neurons and the Rectified Linear Unit (ReLU)^34^ activation function. Last, the outputs emanating from these layers are combined along the feature axis, yielding a single tensor we refer to as *fused drug embeddings* in Figure 1. We thus have a tensor that summarizes the SMILES sequence three distinct ways.

#### Protein Fusion Block

Mirroring the structure of the drug fusion block, the right side of the MDF-DTA architecture in Figure 1 depicts a protein fusion block consisting of three components for extracting protein embeddings. The first component employs ProtVec,^30^ a pretrained model responsible for extracting 1D embeddings with a length of 100 from protein sequences. The next component employs a pretrained ProtBERT model^31^ processes protein sequences, resulting in 2D embeddings with a length of 768. The third component leverages a transformer-based ESM-Fold model,^19^ utilizing a pretrained transformer network to generate length 1280 3D embeddings for protein sequences. L2 normalization is then applied to each component to achieve consistent feature scaling across all embeddings. Each embedding from each of the three components is passed through a fully connected layer that has 1024 neurons implementing ReLU activation. The resulting outputs of these layers are fused together along the feature axis, culminating in a singular tensor we refer to as the *fused protein embeddings* vector.

The fused drug embeddings and fused protein embeddings are further concatenated to generate a tensor. The tensor is fed into a sequence of fully connected layers, defined as
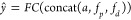
2where *a* denotes the interaction occurring between a protein *f*_*p*_ and a drug *f*_*d*_. To prevent overfitting during training and for regularization, these fully connected layers include dropout layers with a dropout value of 0.3. These fully connected layers consist of three dense layers (*FC*_1_, *FC*_2_, and *FC*_3_), each with 1024, 1024, and 512 neurons, respectively. The model’s output layer has only a single neuron implementing a linear activation, which is often employed for regression tasks to predict numerical values. In training, we strive to minimize the mean squared error (MSE) between ground truth and predicted values.

### Drug Representation

#### SMILES  1D Embedding

The Mol2Vec model^28^ is based on the Word2Vec model,^35^ which is a popular NLP technique used for learning distributed representations of words in a continuous vector space. In a similar manner, Mol2Vec applies this concept to molecular substructures represented in SMILES strings. In the context of Mol2Vec, each SMILES string is treated as a sequence of characters analogous to a sentence in natural language. The model then learns distributed representations (embeddings) for each unique substructure (i.e., atom or functional group) present in the SMILES sequences. We achieve this by training a neural network to predict the surrounding substructures given a target substructure, similar to how Word2Vec predicts the context words when provided with a target word as a part of a sentence.

During training, the Mol2Vec model iteratively adjusts the embeddings of molecular substructures to minimize the prediction error, effectively capturing the relationships and contextual information encoded in the SMILES sequences. It employs a skip-gram algorithm to capture spatial relationships more effectively by considering sequence context weights. As a result, the learned embeddings represent meaningful and semantically rich representations of molecular substructures in a continuous vector space. To obtain 1D embeddings from SMILES sequences using Mol2Vec, the model processes each SMILES string character by character, extracting the embeddings corresponding to individual substructures encountered along the sequence. These embeddings are concatenated to form a single vector representation, resulting in a 1D embedding that captures the features of the entire molecule encoded in the SMILES string. The process can be summarized as tokenizing a molecule represented using SMILES notation and assigning a unique vector representation to each token, denoted as ‘E(token)’. These tokens are then input into a recurrent neural network (RNN) that processes the tokens sequentially, capturing the underlying dependencies and contextual information within the SMILES sequence. This sequence representation can be represented as
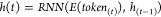
3where *h*_(*t*)_ signifies the RNN’s hidden state at time *t*.

#### SMILES  2D Embedding

To capture the topological features from 2D graphs of the SMILES, we employ a pretrained GIN model,^24^ a GNN-based architecture that operates on graph-structured data. Since the structure of a molecule can be viewed as an undirected graph, with the nodes being the atoms and bonds being the edges. The GIN model was trained using an unsupervised manner on a large data set of molecular graphs. The pretraining task for GINs is graph reconstruction, where the model learns to reconstruct an original molecular graph structure from an input molecular graph. This task encourages the GIN to learn meaningful representations of molecular graphs by capturing important structural features and relationships between atoms and bonds. The GIN model is capable of capturing topological features of graphs through a message-passing mechanism where node embeddings are propagated outward to neighbors, neighbors of neighbors, etc. Then all node embeddings are aggregated and passed through an MLP resulting in an embedding at a particular layer. The GIN model then processes a molecular graph using multiple graph convolutional layers, with each layer consisting of the following steps:

1. **Message Passing**: Each node aggregates information from its neighboring nodes and edges, followed by an update of its own features. Mathematically, the message passing operation at the *l*th layer can be described as
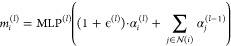
where α_*i*_^(*l*)^ is the feature vector of node *v*_*i*_ at layer *l*, N(*i*) denotes the set of neighboring nodes of node *v*_*i*_, MLP^(*l*)^ is a multilayer perceptron applied element-wise to each node, and ϵ^(*l*)^ is a trainable parameter to capture self-loops in the graph.
2. **Aggregation**: After message passing, the updated node representations are aggregated across all nodes to create a global representation of the graph.
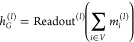
3. **Update**: The global graph representation obtained from the aggregation step is passed through another MLP to generate the final output representation for the graph at layer *l*.
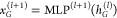

We repeat this process for multiple layers to allow for hierarchical feature extraction and refinement. At the final layer, the output representation *x*_*G*_^(*L*)^ captures the learned features of the entire molecular graph, encoding its topological characteristics. This model represented as *f*_2 D_, takes the adjacency matrix (A), atom attributes (X), and bond attributes (E) as input and generates an embedding vector ***z***_*i*_^2 D^ ∈ *M*^*D*^ for each molecule (M), where *D* represents the dimension of the embedding space. Mathematically, this can be expressed as
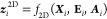
4The resulting embeddings provide a compact representation of the 2D structural features of molecules. These 2D features provide a richer embedding space more so than than 1D features and thus can provide deeper insights into a molecule’s structure.

#### SMILES  3D Embedding

We used a pretrained EGNN^29^ model that was trained on a large-scale, unlabeled data set to generate 3D embeddings. EGNN works in a similar manner as GNN with an extra attribute of 3D spatial positions of the atoms and bonds. In the context of molecular data, the pretraining task for EGNN involve learning equivariant representations of molecular graphs with respect to rotations, translations, and other geometric transformations. This task aims to capture the three-dimensional spatial arrangements of atoms and bonds in molecules while preserving important structural information to generate 3D embeddings. These 3D embeddings aim to capture the structural characteristics of molecules, including the arrangement of atoms and the spatial geometry of the molecule. The 3D embeddings are generated through message-passing over 3D attributes that capture the features from the spatial arrangement of molecular atoms and residues within SMILES sequences. Computing the 3D conformations is itself a part of EGNN model that enables it to capture spatial arrangements of atoms within molecules and provide accurate 3D representations. Specifically, to obtain 3D conformer attributes from the SMILES sequence, we followed similar steps suggested by authors in previous research.^36^ In 3D space, molecular graphs can be formally described by *G* = (*A*, *R*, *X*, *E*), with *A* ∈{0, 1}^*N*×*N*^ being the adjacency matrix denoting edge-connectivity between N nodes (atoms), *R* ∈ *M*^*N*×3^ represents the 3D spatial positions of the atoms, *X* ∈ *M*^*N*×*K*^ represents K dimensional attributes of an atom, and *E* ∈ *R*^*N*×*N*×*D*^ is the tensor that encodes D dimensional attributes of bonds. Each molecule is also associated with a SMILES string *S* = [*s*_*j*_]_*j* = 1_^*C*^, where *C* represents the characters in a SMILES string, which characterizes its chemical structure.

The 3D embeddings obtained from the EGNN model encode detailed spatial information, allowing for a more precise representation of a molecule’s shape, orientation, and surface characteristics. As a result, the 3D embeddings can better capture the structural features relevant to binding sites and binding interactions between molecules, including the arrangement of functional groups and the spatial complementarity between ligands and receptors.

### Protein Representation

#### Protein Sequence  1D Embedding

. The pretrained ProtVec^30^ model was used to produce feature representations for protein sequences in 1D space. With the ProtVec model, sequential information is extracted from protein amino acid sequences. For training, the ProtVec model uses Skip-gram word embeddings in a manner similar to that of the Word2Vec^35^ model. ProtVec makes use of a large corpus of protein sequences to train and generate embeddings for biological targets.

#### Protein Sequence  2D Embedding

. To capture the 2D topological features embedded within protein sequences, we used a pretrained ProtBERT^31^ model. The ProtBERT model is trained on a vast unlabeled data set comprising 1D protein sequences. Although the ProtBERT model takes 1D protein sequence as input, we categorized it under the 2D designation because the embeddings generated by ProtBERT capture information about protein sequences based on the contacts between amino acids in their 2D structures. The protein-encoding layer in ProtBERT uses a transformer^16^ equipped with multihead attention for encoding with input as amino acid sequences and produces a latent encoding of protein amino acid sequences as vectors. An input protein sequence *p* = [*p*_1_,. .., *p*_*n*_], with *p*_*i*_ ∈ 21 being amino acid types, is transformed by the transformer model into a latent representation *l* = [*l*_1_,. .., *l*_*n*_] as shown below:
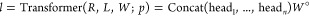
5
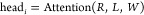
6
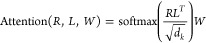
7where *R* ∈ *S*^*d*_1_ × *d*_2_^, *L* ∈ *S*^*d*_1_ × *d*_2_^, *W* ∈ *S*^*d*_1_ × *d*_2_^ represents attention parameters, *n* shows the head count, *W*^*o*^ ∈ *S*^*d*_1_ × *d*_2_^ shows head weight and  represents the Q dimension. Weights on the values are obtained by dividing the self-attention function by a softmax function, which is computed on the dot products of each query with all keys at the same time. It is worth mentioning that we projected the structural embeddings generated by ProtBERT into a lower-dimensional space.

#### Protein Sequence  3D Embedding

To extend the dimensions of our protein sequence embeddings and access their 3D features, we used a transformer-based model named ESM-Fold,^19^ trained on a vast, unlabeled data set, enabling it to encode protein sequences into a tensor of embeddings. Considering the one-hot embedding {*x*_1_^*P*^, ···, *x*_*m*_^*P*^} ∈ ^*V p*^, the ESM-Fold model’s output is described below:
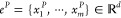
8where *d* denotes the ESM-Fold hidden layer dimension and *V*_*p*_ is the size of the amino acid sequence.

The final representation obtained using this model is a 3D embedding of protein sequences based on the outputs of the last layer of the pretrained ESM-Fold model. In the Experimental Evaluation section, we describe that these embeddings prove to be a critical component of our model as they increase the accuracy of MDF-DTA predictions.

## Experimental Evaluation and Results

### Experimental Setup

Our MDF-DTA model is built using the Keras^37^ framework. We used the scikit-learn library to partition the data sets into train sets (80%) and test sets (20%) and also to ensure consistency in data set splits across all the state-of-the-art models. To train MDF-DTA, an Adam optimizer with a learning rate (η) of 0.0001 is used. MSE^38^ is chosen as the loss function in accordance with the usual approach for regression tasks. We trained for a maximum of 500 epochs with early stopping and with a batch size of 64. To aid with hyperparameter tuning, the training set undergoes an additional 5-fold cross-validation where we split the data into five different subsets. Four of these subsets are used for training, while the fifth subset is used for validation.

As shown in Figure 1, for the 1D, 2D, and 3D dimensions, the resulting drug embeddings have different dimensions, with lengths of 100, 300, and 512, respectively. The latent representation lengths for the 1D, 2D, and 3D embeddings of the proteins are 100, 768, and 1280, respectively. The dense layers in both fusion blocks use 1024 neurons, with the exception of the layers that come after the concatenations to predict the affinity score, which has 512 neurons. To avoid overfitting, dropout regularisation is used at a rate of 0.3 on every dense layer. After concatenation, three fully connected layers are added for affinity prediction. We utilize four different evaluation metrics, including the AUPR,^39^*r*_*m*_^2^,^40^ MSE,^38^ and CI.^41^ In addition, we computed standard deviations for CI, *r*_*m*_^2^, and AUPR that offer important details regarding the model’s predictions. We note that due to the unavailability of standard deviation of MSE metric in the literature, we also presented MDF-DTA results without MSE standard deviation.

### Overall Performance: Predicting Drug-Target Affinity

As presented in Table 1 and Table 2, we used the DAVIS^32^ and KIBA^33^ data sets, respectively, to compare MDF-DTA against current state-of-the-art DTA techniques including KronRLS,^7^ SimBoost,^8^ SimCNN-DTA,^42^ DeepDTA,^9^ WideDTA,^12^ AttentionDTA,^13^ MATT-DTI,^14^ GraphDTA,^23^ TransVAEDTA,^21^ and FusionDTA.^18^ We evaluated the performance of MDF-DTA for both classification tasks and regression tasks. Three common metrics were used to evaluate performance on the regression task: CI, MSE, and *r*_*m*_^2^. For the classification task, MDF-DTA is evaluated using precision-recall, which is represented by AUPR metric. To calculate the AUPR score, we set the threshold values for both data sets as provided in the literature:^9^ a threshold value of 7 for the DAVIS data set and 12.1 for the KIBA data set.

**Table 1:** A Comparative Analysis of the Results Obtained by the MDF-DTA Model in Contrast to Various State-of-the-Art Models on the KIBA Data Set

**Models**	MSE *↓*	**C l***↑*	*r*_*m*_^2^*↑*	AUPR *↑*
KronRLS *(Pahikkala et al., 2015)*(7)	0.411	0.782(0.001)	0.342(0.001)	0.635(0.004)
SimBoost *(He et al., 2017)*(8)	0.222	0.836(0.001)	0.629(0.007)	0.760(0.003)
SimCNN-DTA *(Shim et al., 2021)*(42)	0.274	0.821(0.001)	0.573(0.003)	0.721(0.001)
DeepDTA *(Ozturk et al., 2018)*(9)	0.194	0.863(0.002)	0.673(0.009)	0.788(0.004)
WideDTA *(Ozturk et al., 2019)*(12)	0.204	0.854(0.001)	0.692(0.009)	-
FusionDTA *(Yuan et al., 2022)*(18)	0.167	0.890(0.001)	0.699(0.010)	0.831(0.003)
MATT-DTI *(Zeng et al., 2021)*(14)	0.150	0.889(0.001)	0.756(0.011)	-
GraphDTA *(Nguyen et al., 2021)*(23)	0.162	0.879(0.004)	0.736(0.028)	0.823(0.009)
AttentionDTA *(Zhao et al., 2023)*(13)	0.155	0.882(0.004)	0.755(0.017)	0.829(0.005)
TransVAEDTA *(Zhou et al., 2024)*(21)	0.253	0.822(0.002)	0.632(0.001)	0.701(0.004)
**MDF-DTA**	**0.146**	**0.892**(**0.002**)	**0.787**(**0.005**)	**0.848**(**0.003**)

**Table 2:** A Comparative Analysis of the Results Obtained by the MDF-DTA Model in Contrast to Various State-of-the-Art Models on the DAVIS Data Set

**Models**	MSE *↓*	**C l***↑*	*r*_*m*_^2^*↑*	AUPR *↑*
KronRLS *(Pahikkala et al., 2015)*(7)	0.379	0.871(0.001)	0.407(0.005)	0.661(0.010)
SimBoost *(He et al., 2017)*(8)	0.282	0.872(0.002)	0.644(0.006)	0.709(0.008)
SimCNN-DTA *(Shim et al., 2021)*(42)	0.319	0.852(0.002)	0.595(0.01)	0.657(0.007)
DeepDTA *(Ozturk et al., 2018)*(9)	0.261	0.878(0.004)	0.630(0.017)	0.714(0.010)
WideDTA *(Ozturk et al., 2019)*(12)	0.262	0.886(0.003)	0.633(0.007)	-
FusionDTA *(Yuan et al., 2022)*(18)	0.220	0.903(0.002)	0.666(0.008)	0.773(0.008)
MATT-DTI *(Zeng et al., 2021)*(14)	0.227	0.891(0.003)	0.683(0.009)	-
GraphDTA *(Nguyen et al., 2021)*(23)	0.258	0.884(0.002)	0.656(0.014)	0.710(0.006)
AttentionDTA *(Zhao et al., 2023)*(13)	0.216	0.893(0.005)	0.677(0.024)	0.776(0.024)
TransVAEDTA *(Zhou et al., 2024)*(21)	0.332	0.869(0.008)	0.571(0.001)	0.662(0.003)
**MDF-DTA**	**0.172**	0.912(0.003)	**0.763**(0.009)	**0.792**(**0.004**)

To compare with existing DTA models, we first provide the experimental results on the KIBA data set.^33^Table 1 provides a summary of the overall performance results of MDF-DTA with different state-of-the-art models with the previously discussed metrics. Our experimental results show MDF-DTA’s outstanding performance on the KIBA data set. MDF-DTA outperforms all other approaches, doing exceptionally well on each of the evaluation metrics for both regression and classification tasks. In the context of regression, an improvement in the CI index is observed with a value of 0.892, when compared to the best state-of-the-art model. Additionally, MDF-DTA obtains an MSE value of 0.146, less than the best MSE value that baseline models achieve. MDF-DTA shows an improved 0.787 when compared to the best-performing MATT-DTI^14^ for the *r*_*m*_^2^ metric. Our model shows an improved 0.848 AUPR score in the classification task.

**Figure 3:**
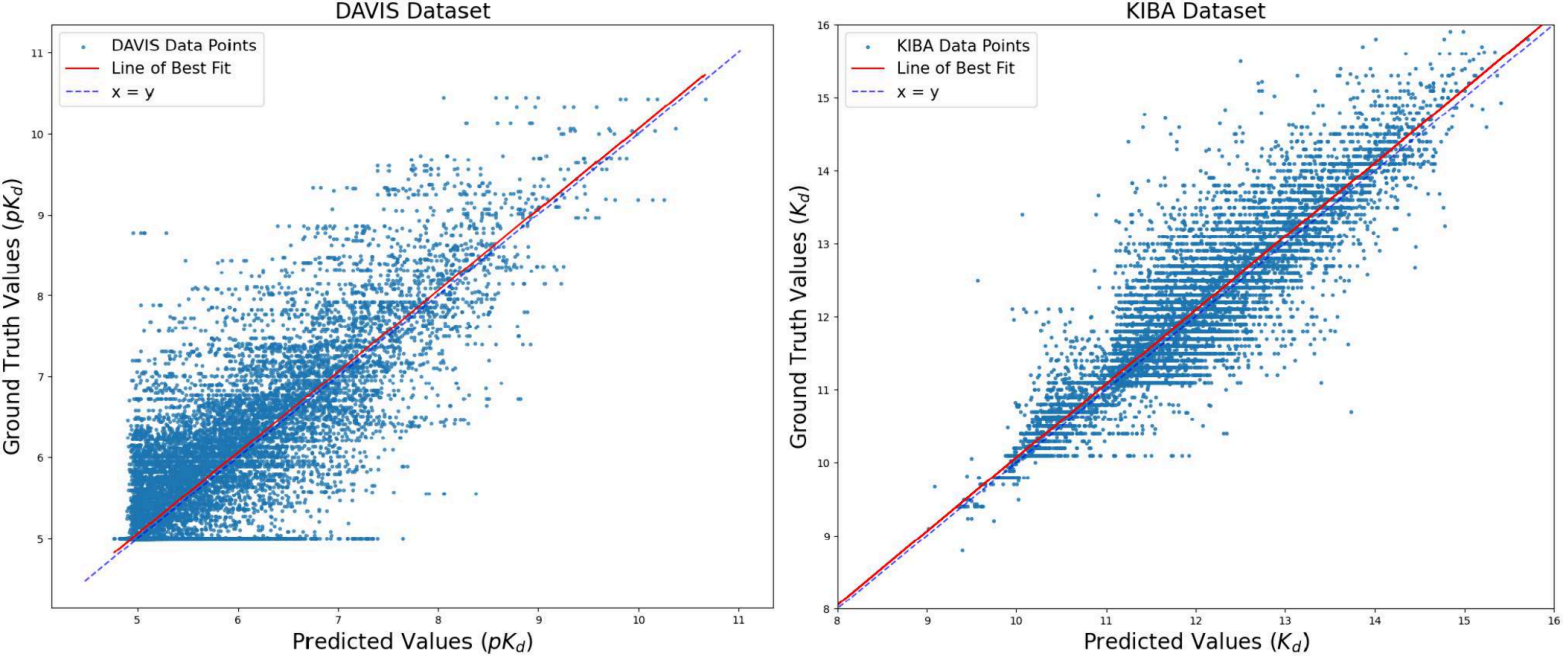
Scatterplots comparing predicted (horizontal axis) versus ground truth affinities (vertical axis) for the DAVIS Data set (left) and the KIBA Data set (right). The residual squared in the scatter plots between the predicted and ground truth value is the vertical distance |*y* – *ŷ*| from each data point.

We then evaluated MDF-DTA’s performance on the DAVIS^32^ data set by assessing its results with those of baseline models. Table 2 summarizes the comparative results. On the DAVIS data set, MDF-DTA performs better than baseline models with respect to MSE, CI, *r*_*m*_^2^ and AUPR. In particular, the MSE value obtained by MDF-DTA is 0.172, which is much lower than that of the best-performing baseline model. With respect to the CI metric, MDF-DTA outperforms FusionDTA, the best baseline model, by stating 0.912 higher. Furthermore, MDF-DTA improves over MATT-DTI^14^ and outperforms baseline models in the *r*_*m*_^2^ index with a value of 0.763. These results demonstrate that MDF-DTA can precisely predict affinity scores with better accuracy. Also, our model outperforms each baseline model in the binary classification task, achieving the greatest AUPR score, with a peak value of 0.792. For the DAVIS and KIBA data sets, we provide a visual representation of the predicted and real affinity values through scatter plots in Figure 3. As shown in Figure 2, most affinity scores for the DAVIS and KIBA data sets lie in the ranges of 5 to 10 and 9 to 16, respectively. Consequently, in the case of predicted values, they also fall within the same ranges, as illustrated in Figure 3. In Figure 3, the horizontal axis represents predicted affinity values, and the vertical axis corresponds to ground truth values. The squared difference between the predicted output and the ground truth value is the vertical distance |*y* – *ŷ*| calculated from each data point. For both the DAVIS and KIBA data sets, it can be observed that the data points generally show symmetry around the best-fit line. In particular, the KIBA data set data points are more widely scattered around the best-fit line.

MDF-DTA’s *r*_*m*_^2^ values of 0.787 and 0.763 for the two data sets validate the model’s ability to fit and align with real data accurately. In addition, for each data set, the McNemar test^43^ was performed to understand the statistical significance of the improvements provided by MDF-DTA compared to the best-performing state-of-the-art model, AttentionDTA. Table 3 presents the results of McNemar’s test showing that improvements among all the metrics as obtained from MDF-DTA compared with AttentionDTA are statistically significant, with p-values <0.05. This is further evidence of the effectiveness of our proposed MDF-DTA in predicting affinity scores.

**Table 3:** Statistical Analysis Showing the p-Values for the McNemar Test for Understanding the Statistical Significance of the Improvements Provided by MDF-DTA as Compared to AttentionDTA

Data set	MSE	CI	*r*_*m*_^2^	AUPR
DAVIS	5.7 × 10^–3^	2.9 × 10^–2^	7.7 × 10^–3^	1.9 × 10^–3^
KIBA	7.3 × 10^–5^	4.3 × 10^–2^	3.5 × 10^–2^	3.2 × 10^–4^

### Ablation Studies

In this section, we examine the impact of our multidimensional fusion approach for computing DTA. Our objective is 2-fold: to determine the benefit of adding embeddings associated with drugs and proteins and to analyze the individual contributions of each dimensional embedding type. To accomplish this task, we perform a sequence of ablation experiments in which we systematically add and remove dimensional embedding and analyze the impact on the prediction power of the model.

We first extract and evaluate the unique input of each dimensional embedding that is included in the MDF-DTA model. The results of removing one embedding at a time are shown in Table 4. We observe that each dimensional embedding is essential in the model’s performance: the overall model is more than just the sum of its parts. Remarkably, removing the ESM-Fold (3D Target) target embedding and the EGNN (3D) drug embedding results in a significant change in model performance, indicating the presence of unique information contained within both embeddings. We also observed that the simple concatenation of EGNN and ESMFold embeddings performs better in some scenarios compared to excluding a few of the embeddings from MDF-DTA on the KIBA data set. This is because simple concatenation works well when utilizing a single representation of drugs and proteins, especially when the data set is significantly large. However, it fails when the data set is small, as in the case of the DAVIS data set. Additionally, simple concatenation struggles when multiple representations of drugs and proteins are used to predict affinity scores. On the other hand, MDF-DTA performs better in handling multiple representations of drugs and proteins on both the KIBA and DAVIS data sets. In particular, in the DAVIS and KIBA data sets, the removal of EGNN causes an increase in MSE to 0.209 and 0.177, respectively. In the same data sets, the removal of the ESM-Fold embedding causes an increase in MSE to 0.218 and 0.185, respectively. These results show the roles that the EGNN (3D drug) and ESM-Fold (3D target) embeddings play in the prediction of drug-target interactions. However, while the ProtBERT and ProtVec target embeddings give slightly less unique information than the EGNN and ESM-Fold, they still have a significant impact. In the DAVIS and KIBA data sets, removing the target embedding ProtBERT causes an MSE rise to 0.204 and 0.174, respectively. With regard to drug-target interaction prediction, these ablation studies amply illustrate the diversity of features and the importance of various embeddings. Among the embeddings investigated, EGNN for drugs and ESM-Fold for proteins stand out as the most important, containing unique and valuable data. In contrast, the MDF-DTA performance does not change much when either of the two retained embeddings, the ESM-Fold target embedding or the EGNN drug embedding is used. When the EGNN drug embedding and the ESM-Fold target embedding are separated, the MSE changes significantly.

**Table 4:** Results of an Ablation Analysis, in Which Each Dimensional Embedding Is Systematically Excluded from MDF-DTA for Both the KIBA and DAVIS Data Sets

	**KIBA**				**DAVIS**			
**Embedding Excluded**	MSE *↓*	CI *↑*	*r*_*m*_^2^*↑*	AUPR *↑*	MSE *↓*	CI *↑*	*r*_*m*_^2^*↑*	AUPR *↑*
EGNN	0.177	0.878	0.758	0.830	0.209	0.879	0.672	0.770
GIN	0.168	0.879	0.775	0.832	0.198	0.880	0.687	0.772
Mol2Vec	0.149	0.891	0.784	0.845	0.176	0.892	0.695	0.784
ESMFold	0.185	0.875	0.749	0.831	0.218	0.876	0.664	0.771
ProtBert	0.174	0.887	0.774	0.839	0.204	0.888	0.686	0.778
ProtVec	0.148	0.879	0.782	0.843	0.174	0.891	0.693	0.782
**MDF-DTA**	**0.146**	**0.892**	**0.787**	**0.848**	**0.172**	**0.912**	**0.763**	**0.792**

Our next step in the ablation study is to assess the model’s performance when considering only a single dimension for either the drug or the target. For this purpose, we used simple concatenation of embeddings for both the drug and the protein instead of using the fusion approach, as fusion did not make any sense in this scenario. The concatenated tensor passes through three fully connected layers to obtain a prediction of the final affinity score. The results obtained from the KIBA and DAVIS data sets are shown in Table 5, and they provide important insights into the capability of individual embedding for drug-target interaction prediction. Surprisingly, when some embeddings, such as Mol2Vec, GIN, ProtVec, and ProtBERT, are used as input for either drugs or targets, the model’s performance tends to be poor. It indicates that these embeddings possess comparatively less useful information to predict drug-target interactions, and their removal does not significantly alter the performance of the model. In fact, in the DAVIS data set, our model’s MSE dramatically drops to 0.303 when Mol2Vec and ProtVec are used as the only input.

**Table 5:** Ablation-Based Performance Evaluation^a^

	**KIBA**				**DAVIS**			
**SMILES** + **Protein**	MSE *↓*	CI *↑*	*r*_*m*_^2^*↑*	AUPR *↑*	MSE *↓*	CI *↑*	*r*_*m*_^2^*↑*	AUPR *↑*
1D + 1D	0.250	0.822	0.634	0.739	0.303	0.878	0.614	0.687
1D + 2D	0.201	0.846	0.703	0.789	0.278	0.884	0.630	0.678
1D + 3D	0.186	0.853	0.715	0.803	0.264	0.887	0.648	0.701
2D + 1D	0.254	0.820	0.636	0.738	0.292	0.876	0.616	0.705
2D + 2D	0.233	0.837	0.661	0.764	0.277	0.883	0.629	0.711
2D + 3D	0.216	0.839	0.686	0.774	0.262	0.889	0.645	0.706
3D + 1D	0.298	0.800	0.564	0.696	0.288	0.875	0.615	0.693
3D + 2D	0.169	0.867	0.729	0.844	0.270	0.884	0.644	0.718
3D + 3D	0.151	0.878	0.782	0.843	0.261	0.892	0.651	0.721
**MDF-DTA**	**0.146**	**0.892**	**0.787**	**0.848**	**0.172**	**0.912**	**0.763**	**0.792**

a As we have three dimensions of embeddings for both drugs and protein target sequences, we evaluate all 9 combinations of size 2, thus including only a particular embedding for both drug and protein target. For example, 1D + 2D is interpreted as only a 1D drug embedding and 2D protein embeddings are included. Analysis is performed on the KIBA and DAVIS data sets.

## Conclusions

In our study, we introduced the novel MDF-DTA model for predicting the affinity score between drugs and target proteins. Our proposed approach provides an improved technique for determining the binding affinity scores among drugs and target proteins. We achieved this by using different types of embeddings having multiple dimensions for both the drugs and proteins. Our experimental results show that certain types of embeddings, particularly EGNN drug representation and ESM-Fold target representation, play a major role in accurate affinity predictions. Through the combination of three drug embeddings and three target embeddings, together with their related network encoding, our model provides a comprehensive understanding of the roles played by each embedding in binary classification and regression tasks. MDF-DTA outperforms single embedding tests, demonstrating the effectiveness of multidimensional fusion approach. In our study, we have captured high-level representations of interactions among molecular substructures and constituent atoms through the use of multidimensional embeddings for both drugs and target proteins. By employing pretrained models, we aim to capture diverse structural and spatial information at various levels of granularity. These embeddings encode information about molecular substructures, topological features, and spatial arrangements of atoms, which indirectly reflect the underlying interactions between molecular components. Additionally, our model architecture incorporates fusion blocks for both drugs and proteins, allowing for the integration of information from multiple sources. This enables the model to leverage the collective knowledge encoded in different types of embeddings and learn complex interactions between molecular entities. Additionally, the ablation studies illustrate the special qualities of each embedding and the importance of the ESM-Fold protein and the E3NN drug embedding.

Since MDF-DTA used a pretrained model to generate the embeddings, this lowers the overall computational cost of training the models. Our choice of using a simpler model, the Multi-Layer Perceptron (MLP) with dropout layers for affinity prediction, offers several advantages, including interpretability, requiring fewer parameters for effective training, faster training time, and robustness to overfitting. In fields such as healthcare, understanding the decision-making process is as crucial as the decision itself, and the interpretability of simpler models like MLPs contributes significantly to this understanding compared to conventional machine learning algorithms. We also believe that interpretability is indeed a crucial aspect in the deployment of predictive models for DTA prediction, particularly when used for virtual screening of drugs. We considered interpretability in terms of how different molecular features contribute to the overall prediction of drug-target affinity. In this study, our focus has primarily been on improving the accuracy and performance of DTA prediction through our proposed MDF-DTA model.

Despite the fact that our MDF-DTA model obtained a substantial improvement in computational affinity prediction on benchmark data sets and represents a step toward capturing how molecular substructures and constituent atoms interact, we acknowledge that there is still room for improvement. Future research directions could include the development of more sophisticated models that explicitly model these interactions or the incorporation of additional features that capture atomic-level interactions. Also, in future iterations of our model, we will incorporate testing on a wider range of protein categories and explore additional techniques to enhance interpretability. This includes conducting docking experiments to demonstrate the amino acid residues surrounding the binding pocket of the complex as well as illustrating the intermolecular bonds responsible for the optimal ligand pose. These efforts aim to provide biologically plausible cues for experts to understand the drug-target interaction mechanism better. Furthermore, our goal is to be good at designing a better deep-learning model and making these advances practical for the drug discovery pipeline. Additionally, exploring deeper, more complex interaction blocks, incorporating recent advances like self-attention, and assessing the model’s architecture against state-of-the-art fusion architectures are key considerations for further research. The ultimate goal is to deploy DTA as a service that can not only prodce accurate predictions the affinity score between drugs and target proteins but can also provide the rationale behind such predictions.

## Data Availability

All the source code and data are made available under the MIT License at https://github.com/Amitranjan71/MDF-DTA.git. Since the obtained embeddings from the benchmark data sets are large, they can be made available upon request.
